# miR‐205‐3p promotes proliferation and reduces apoptosis of breast cancer MCF‐7 cells and is associated with poor prognosis of breast cancer patients

**DOI:** 10.1002/jcla.22966

**Published:** 2019-10-02

**Authors:** Changhong Qiu, Fei Huang, Qing Zhang, Wei Chen, Huiting Zhang

**Affiliations:** ^1^ Department of General Surgery The First People's Hospital of ZhaoQing ZhaoQing China; ^2^ Department of General Surgery The Seventh Affiliated Hospital of Sun Yat‐Sen University (Shen Zhen) Shen Zhen China

**Keywords:** apoptosis, breast cancer, miR‐205‐3p, prognosis, proliferation

## Abstract

**Background:**

To study the expression of microribonucleic acid (miR)‐205 in breast cancer and its effects on the proliferation and apoptosis of breast cancer cells.

**Methods:**

Breast cancer cell line MCF‐7 cells with stable expression of miR‐205‐3p were constructed. Cell proliferation, invasion, and apoptosis were detected via MTT assay, transwell assay, and flow cytometry, respectively. The expressions of Ezrin, LaminA/C, cleaved caspase‐3, Bcl‐2, and Bax were detected via Western blotting. The expressions of miR‐205‐3p in breast cancer tissues and para‐carcinoma tissues were detected via quantitative PCR (qPCR).

**Results:**

In transfection group, cell proliferation and invasion capacities were increased significantly (*P* < 0.01), but apoptotic cells were significantly reduced (*P* < 0.01). In addition, the expressions of Ezrin, LaminA/C, and cleaved caspase‐3 in the transfection group were significantly decreased (*P* < 0.01), but the Bcl‐2/Bax ratio was significantly increased (*P* < 0.01). The miR‐205‐3p expression in tumor tissues of breast cancer patients was significantly higher than that in para‐carcinoma tissue, but Ezrin, LaminA/C, and cleaved caspase‐3 expressions in tumor tissues were remarkably declined (*P* < 0.01), while the Bcl‐2/Bax ratio was remarkably increased (*P* < 0.01). Moreover, the 5‐year survival of patients with high expression of miR‐205‐3p was significantly shorter than patients with normal or low expression (*P* < 0.01).

**Conclusion:**

Highly expressed miR‐205‐3p can promote the proliferation and invasion and reduce the apoptosis of breast cancer cells, and the high expression of miR‐205‐3p can significantly reduce the survival time of patients.

## 1 INTRODUCTION

Both morbidity and mortality rates of breast cancer, as one of the common malignant tumors in women, are ranked 1st in a variety of female tumors, seriously threatening the female health^1^ The number of breast cancer patients diagnosed and treated each year is not large in China compared with that in Western developed countries, but the incidence rate of breast cancer has displayed an increasing trend in the last 2 decades. In particular, breast cancer has become the malignant tumor with the highest incidence rate in women in big cities^2^ A variety of stress responses in cells will be produced when various external stimuli (various carcinogenic factors) act on breast epithelial cells, resulting in cell proliferation, thus leading to breast cancer^3^ At present, surgical operation is dominated in the clinical treatment approaches of breast cancer, but the range of surgical resection in clinic is wide, the trauma is large, and a lot of related complications often occur in patients after clinical surgical treatment, causing serious physiological and psychological damage to patients^4^.

A large number of studies in recent years have revealed that various specific gene mutations were identified in tumor patients and several microribonucleic acids (miRNAs) are closely related to the tumor metastasis and invasion^5^, ^6^ Osipov et al^7^ found high expression of miR‐205 in epithelial ovarian cancer cells, indicating that miR‐205 is possibly associated with the incidence of ovarian cancer. There is a great amount of research evidence proving that the miR‐205 expressions in a variety of tumors are lower than that in normal tissues^8^, ^9^, ^10^, ^11^ miRNAs are therefore applied in the diagnosis of cancer^12^, ^13^ For instance, miR‐205 presents as the potential biomarkers for early prognosis or diagnosis while its level is proposed in predicting lymph node metastasis in triple‐negative breast cancer patients^14^, ^15^ Moreover, it has been demonstrated that miR‐205/ASPP2 axis promoted cell migration and also increased cell proliferation, which may be potential diagnostic and therapeutic biomarkers in cervical and lung cancers^16^ miR‐205 enhances chemosensitivity of breast cancer cells to TAC chemotherapy by suppressing both VEGFA and FGF2, leading to evasion of apoptosis^17^ However, the exact role of miR‐205‐3p in the pathogenesis and development of breast cancer remains poorly understood. The present study aims to investigate the role and possible mechanism of miR‐205‐3p in breast cancer, in order to provide a theoretical basis for further elaborating the effect of miR‐205‐3p in the course of breast cancer.

## 2 PATIENTS AND METHODS

### 2.1 Patients and grouping

A total of 58 patients who were pathologically diagnosed with breast cancer, treated, and underwent mastectomy in Guangdong Provincial People's Hospital from June 2011 to June 2013 were collected. None of the patients received chemotherapy and radiotherapy before operation. The para‐carcinoma tissues of breast cancer (more than 5 cm away from the breast cancer tissues) were taken during operation as the control. All enrolled patients were females aged 42‐69 years with a median age of 56.2 years, and they all suffered from invasive ductal carcinoma. According to the tumor‐node‐metastasis (TNM) staging criteria issued by the International Union Against Cancer, patients were staged, and there were 8 cases in stage I, 41 cases in stage II, 6 cases in stage III, and 3 cases in stage IV. Inclusion criteria were as follows: (1) patients without severe hepatic and renal function impairment, (2) patients who agreed to be followed up, (3) patients without a history of serious cardiovascular and immune system diseases, and (4) patients without chronic or acute infectious diseases. All enrolled patients personally signed the informed consent and had complete clinical and pathological data and complete therapeutic regimens. The experimental scheme was reviewed and approved ethically by our hospital.

### 2.2 Detection of miR‐205‐3p expression level in breast cancer tissues

After breast cancer tissues and para‐carcinoma tissues were obtained, they were added into the tube (100 mg/mL) together with TRIzol, and smashed using an ultrasonic disruptor followed by being incubated on ice for 10 minutes, followed by centrifugation at 12 000 *g* and 4°C for 10 minutes. 700 μL supernatant was taken, added with chloroform, and manually vibrated for 10 times, followed by incubation on ice for 10 minutes and centrifugation at 12 000 *g* and 4°C for 10 minutes. After the supernatant was discarded, 1 mL freshly prepared 75% ethanol was added into each tube, followed by centrifugation at 12 000 *g* and 4°C for 15 minutes. The ethanol was discarded, the cap of the centrifuge tube was removed for natural air‐drying, and 30 μL diethyl pyrocarbonate (DEPC)‐treated water was added to dissolve the RNA. The optical density (OD) value and purity of RNA were measured. The reaction system was prepared strictly according to instructions of the reverse transcription kit (Invitrogen): 5 μg total RNA, 1 μL Oligo(dT)_20_, 1 μL 10 mmol/L dNTP, and 3 μL 0.1mol/L DTT were added, and DEPC‐treated water was also added until the total volume was 20 μL. After reaction at 60°C for 15 minutes and incubation at 37°C for 2 minutes, the reverse transcription was performed. After RT‐qPCR system was prepared, the amplification was performed on the RT‐qPCR instrument. Amplification conditions are as follows: pre‐denaturation at 92°C for 2 minutes, denaturation at 95°C for 20 seconds, annealing at 60°C for 40 seconds, extension at 72°C for 2 minutes, and a total of 35 cycles. Primers were synthesized by Invitrogen, and RNU6B was used as the internal control. The primer sequence was as follows: 5′‐CGG GAT TTC AGT GGA GTG AAG TTC‐3′ (miR‐205‐3p); 5′‐CTC GCT TCG GCA GCA CA‐3′ (RNU6B, sense) and 5′‐AAC GCT TCA CGA ATT TGC GT‐3′ (RNU6B, antisense). The expression of miR‐205‐3p was quantified as a relative expression to RNU6B using 2^−△△^CT method. All RT‐qPCR experiments were conducted according to the MIQE (minimum information for publication of quantitative real‐time PCR experiments) guidelines^18^ Each amplification reaction was performed in triplicate, and mean value of the three‐cycle threshold was used for further analysis. Based on the median expression of miR‐205‐3p, patients were further divided into high and low expression group.

### 2.3 Survival curve analysis

The miR‐205‐3p expression in cancer tissues of each patient with breast cancer was detected and compared with that in para‐carcinoma tissue, and divided into normal or low expression group and high expression group. Data of patients were recorded in detail, and patients were followed up for 5 years. The 5‐year survival rate of patients was recorded, and the survival curve was drawn. Influencing factors of patient's prognosis in both groups were analyzed via Cox multivariate regression analysis.

### 2.4 Cell culture and construction of cell lines with miR‐205‐3p overexpression

Human breast cancer MCF‐7 cell lines were purchased from the Kunming Cell Bank of the Chinese Academy of Sciences. After resuscitation, cells were cultured using Dulbecco's modified Eagle medium (DMEM) (Gibco) containing 10% fetal bovine serum (FBS) in an incubator with 5% CO_2_ at 37°C till the logarithmic growth phase. Then, cells were digested with trypsin and continued to be cultured, followed by passage till the 3rd generation used for the experiment. The gene sequence (5′‐GAUUUCAGUGGAGUGAAGUUC‐3′) of hsa‐miR‐205‐3p was found in the Sanger microRNA database, and primers were synthesized by Invitrogen: forward primer: 5′‐GAGGATCCCCGGGTACCGGTAGGCCTTT‐3′ and reverse primer: 5′‐CACACATTCCACAGGCTGCTACGGTGGTGGCGGCGGGCGGT‐3′. After amplification and electrophoretic separation using agarose gel, the target band was recycled. The GV369 vector (Shanghai GeneChem Co., Ltd.) was cut via double digestion, and the target fragment (5′‐GAUUUCAGUGGAGUGAAGUUC‐3′) was connected to the vector using T4 ligase (Takara), followed by plate clone using LB solid medium at 37°C and sequencing identification using positive colonies. The miR‐205‐3p lentivirus was packaged with the plasmid (TransGen), the titer was detected, and the miR‐205‐3p lentivirus was used to infect MCF‐7 cells after meeting requirements: After digestion, MCF‐7 cells were inoculated into a 24‐well plate at a density of 5 × 10^4^/well. After culturing for 24 hours, 440 μL DMEM, 10 μL hsa‐miR‐205‐3p, and 50 μL polybrene were added into the centrifuge tube, mixed evenly, and inoculated into the plate. After culturing for 12 hours, the original medium was replaced with DMEM containing 10% FBS for culture for another 72 hours. Then, the fluorescence intensity of cells was observed under a microscope. After cells were screened using puromycin (Sigma) and cloned, the MCF‐7 cell lines with stable infection were constructed. Overexpression of miR‐205‐3p was then confirmed by real‐time quantitative polymerase chain reaction (RT‐qPCR) using RNU6B as an internal control.

### 2.5 Detection of cell proliferation and invasion capacities

Cell proliferation capacity was detected via methyl thiazolyl tetrazolium (MTT) assay: After cells in the logarithmic growth phase were digested, the cell density was adjusted to 1 × 10^4^/well, and cells were inoculated into a 96‐well plate and divided into blank control group (MCF‐7 cells without any treatment), negative control group (MCF‐7cells inoculated with empty vector), and hsa‐miR‐205‐3p group (MCF‐7 cells inoculated with hsa‐miR‐205‐3p), followed by culture in an incubator for 24 hours. Then, 100 μL MTT was added to culture cells for another 4 hours, the supernatant was discarded, and 150 μL dimethyl sulfoxide (DMSO) was added into each well and vibrated on a shaking table for 1 minute. Finally, cell proliferation in each group was detected using a microplate reader.

Cell invasion capacity was detected via transwell assay: After cells in the logarithmic growth phase were digested, the cell density was adjusted to 1.5 × 10^6^/well, and the cell suspension was prepared and divided into blank control group (MCF‐7 cells without any treatment), negative control group (MCF‐7 cells inoculated with empty vector), and hsa‐miR‐205‐3p group (MCF‐7 cells inoculated with hsa‐miR‐205‐3p). 300 μL cell suspension was added into the upper side of polycarbonate transwell filter (Corning) containing 50 μL mixture of Matrigel (BD Biosciences) and DMEM (1:4), while 700 μL DMEM containing 10% FBS was added into the lower chamber, followed by culture in the incubator for 24 hours. After being removed from the chamber, cells were fixed with methanol and stained with crystal violet. The cell invasion was observed under the microscope, and the number of cells passing through the membrane was calculated.

### 2.6 Detection of apoptosis

Apoptosis was detected via flow cytometry using FITC Annexin V Apoptosis Detection Kit (BD Bioscience): After cells in the logarithmic growth phase were digested, the cell density was adjusted to 2 × 10^6^/well, and the cell suspension was prepared and divided into blank control group (MCF‐7 cells without any treatment), negative control group (MCF‐7 cells inoculated with empty vector), and hsa‐miR‐205‐3p group (MCF‐7 cells inoculated with hsa‐miR‐205‐3p). Then, the cell suspension was inoculated into a 6‐well plate and added with serum‐free DMEM, followed by culture in the incubator for 24 hours. After the culture solution was discarded, cells were washed with phosphate‐buffered saline (PBS), scraped off, centrifuged, and resuspended with PBS for 3 times strictly according to instructions of the apoptosis kit (BD Bioscience). The fluorescence maker was added and cells were incubated in a dark place at room temperature for 15 minutes, followed by sample loading and detection via flow cytometry. All samples should be detected within 1 hour to ensure the effect.

### 2.7 Detection of expression levels of related proteins

Cells in each group were cultured in the 6‐well plate, collected, followed by addition of radioimmunoprecipitation assay (RIPA) lysis solution (Wuhan Servicebio) and 1% protease inhibitor (Wuhan Servicebio) into each well (500 μL/well). After breast cancer tissues and para‐carcinoma tissues were obtained, they were added with RIPA lysis solution and 1% protease inhibitor (100 mg/mL) and smashed using the ultrasonic disruptor. The above cell suspension and tissue homogenate were incubated on ice, followed by centrifugation at 1000 *g* and 4°C for 10 minutes to obtain the total protein solution. The total protein concentration in each sample was detected using the BCA protein assay kit (PIERCE). After the protein loading buffer in an equal concentration was prepared, 10% gel was prepared and added with an equal amount of loading buffer, followed by sodium dodecyl sulfate polyacrylamide gel electrophoresis under constant pressure. Then, the protein was transferred onto a PVDF membrane (Millipore), and the target band was cut and sealed with freshly prepared 5% skim milk powder for 2 hours. Ezrin, LaminA/C, cleaved caspase‐3, Bcl‐2, Bax, and glyceraldehyde‐3‐phosphate dehydrogenase (GAPDH) antibodies (purchased from CST, USA, and prepared at a volume ratio of 1:1000) were incubated at 4°C overnight. After the band was washed with PBS with Tween‐20 (PBST) for 3 times (5 minutes per time), it was incubated with the horseradish peroxidase‐conjugated secondary antibody (1:5000, Shanghai YIHYSON Biotechnology Co., Ltd.) at room temperature for 1 hour, and then the membrane was washed with Tris‐buffered saline with Tween‐20 (TBST), followed by image development in a dark room: Enhanced chemiluminescence (ECL) solution was added, and images were obtained using the fluorescence imaging technique. The expression level of each protein was presented as Ezrin/GAPDH, LaminA/C/GAPDH, cleaved caspase‐3/GAPDH, and Bcl‐2/Bax, respectively.

### 2.8 Statistical analysis

In this study, three independent experiments were performed for in vitro assay. Data were presented as mean ± SD and processed using Statistical Product and Service Solutions (SPSS) 19.0 software (SPSS Inc.). Paired Student's *t* test was used for the comparison between two groups, chi‐square test was used for enumeration data, and analysis of variance was used for the comparison among groups. The homogeneity test of variance was performed, and Bonferroni's method was used for the pairwise comparison in case of homogeneity of variance, while Welch's method was used in case of heterogeneity of variance. The Pearson correlation analysis was adopted for correlation analysis. Cox proportional hazards model was performed for multivariate analysis of the independent factors for the survival of breast cancer patients. *P* < 0.05 suggested that the difference was statistically significant.

## 3 RESULTS

### 3.1 miR‐205‐3p expression in breast cancer tissues

**Figure 1:**
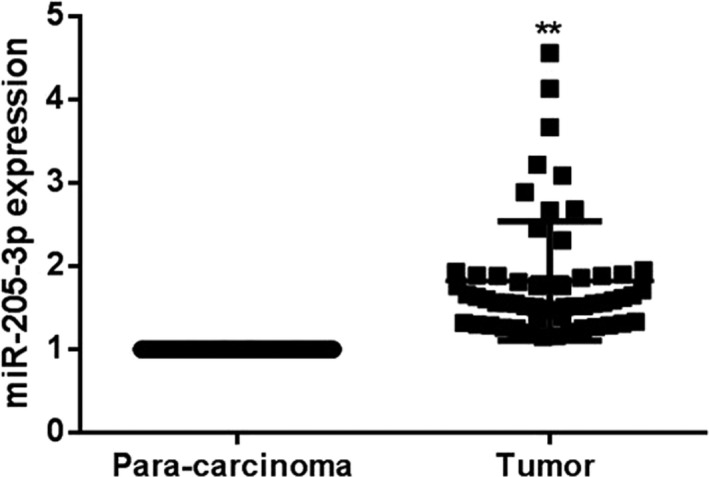
Detection of miR‐205‐3p expressions in tumor tissues and para‐carcinoma tissues in each group via RT‐qPCR. The miR‐205‐3p expression in tumor tissues of breast cancer patients is obviously higher than that in para‐carcinoma tissues, ^**^ *P* < 0.01

The miR‐205‐3p expressions in tumor tissues and para‐carcinoma tissues of breast cancer patients were detected via RT‐qPCR. According to results, the miR‐205‐3p expression in tumor tissues of breast cancer patients was increased significantly compared with that in para‐carcinoma tissues (1.87 ± 0.63 vs 1, *P* < 0.01) (Figure 1).

### 3.2 Related protein expressions in breast cancer tissues

**Figure 2:**
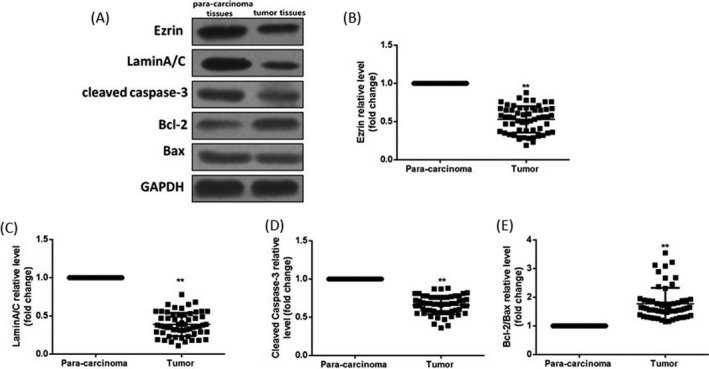
Detection of related protein expressions in breast cancer tissues and para‐carcinoma tissues via Western blotting. (A) Protein bands. (B) Statistical graph of Ezrin protein expression level. (C) Statistical graph of LaminA/C protein expression level. (D) Statistical graph of cleaved caspase‐3 protein expression level. (E) Statistical graph of Bcl‐2/Bax protein expression level. Compared with those in para‐carcinoma tissues, Ezrin, LaminA/C, and cleaved caspase‐3 expressions are remarkably lower in breast cancer tissues, but the Bcl‐2/Bax ratio is higher. Data were presented as a fold change relative to GAPDH (mean ± SD). ^**^ *P* < 0.01

Ezrin, LaminA/C, cleaved caspase‐3, Bcl‐2, and Bax protein expressions in tumor tissues and para‐carcinoma tissues of breast cancer patients were detected via Western blotting. Results manifested that compared with those in para‐carcinoma tissues, Ezrin, LaminA/C, and cleaved caspase‐3 expressions were significantly declined in tumor tissues (*P* < 0.01), but the Bcl‐2/Bax ratio was increased significantly (*P* < 0.01) **(**Figure 2).

### 3.3 Correlations of miR‐205‐3p with clinicopathological features of breast cancer patients

The miR‐205‐3p level was classified as high or low in relation to the median value (cutoff value = 1.704). The above patients were accordingly divided into low or normal expression group and high expression group based on the miR‐205‐3p expression level in breast cancer tissues, with para‐carcinoma tissues as the control. The clinicopathological data of patients in each group were recorded in details, and correlations of miR‐205‐3p expression level with clinicopathological data of patients were analyzed. Results showed that miR‐205‐3p was not related to the age and menstrual condition of breast cancer patients, but statistically closely related to the size, TNM staging, lymph node metastasis, and recurrence of breast cancer (*P* < 0.05) (Table 1). Further univariate and multivariate analysis showed that miR‐205‐3p expression was an independent factor for the survival (recurrence‐free survival) of breast cancer patients (Table 2).

**Table 1:** Clinicopathological data of breast cancer patients

Groups	n	miR‐205‐3p expression level		*χ^2^*	*df*	*P* value	Contingency coefficient
		Low or normal expression	High expression				
Age							
≥50 y old	46	25	21	0.0611	1	0.8047	0.2472
<50 y old	12	7	5				
Menstrual condition							
Premenopause	38	20	18	0.2877	1	0.5917	0.5363
Postmenopause	20	12	8				
Tumor size							
≥2 cm	16	6	10	13.48	1	0.0002	3.672
<2 cm	42	36	6				
TNM staging							
Stages I and II	49	32	17	9.106	1	0.0025	3.108
Stages III and IV	9	1	8				
Lymph node metastasis							
Yes	22	7	15	5.625	1	0.0177	2.372
No	36	23	13				
Recurrence							
Yes	25	7	18	8.507	1	0.0035	2.917
No	33	22	11				

**Table 2:** Multivariate analysis of clinicopathological factors for overall survival (Cox proportional hazards model)

Factors	Univariate analysis		Multivariate analysis	
	HR (95% CI)	*P* value	HR (95% CI)	*P* value
Age (≥50 y)	1.08 (0.43‐2.61)	0.796	/	/
Gender	0.53 (0.21‐1.63)	0.195	/	/
Tumor size (≥2 cm)	6.90 (2.36‐38.2)	0.006	10.6 (0.51‐567)	0.092
TNM staging (III‐IV/I‐II)	1.65 (1.35‐1.93)	0.021	2.76 (0.05‐236)	0.693
Lymph node metastasis (yes/no)	1.03 (1.01‐1.21)	0.031	1.16 (0.15‐25.6)	0.961
miR‐205‐3p expression (high/low)	26.7 (18.3‐45.7)	<0.0001	16.5 (2.61‐359)	0.002

### 3.4 Correlation of miR‐205‐3p with prognosis of breast cancer patients

**Figure 3:**
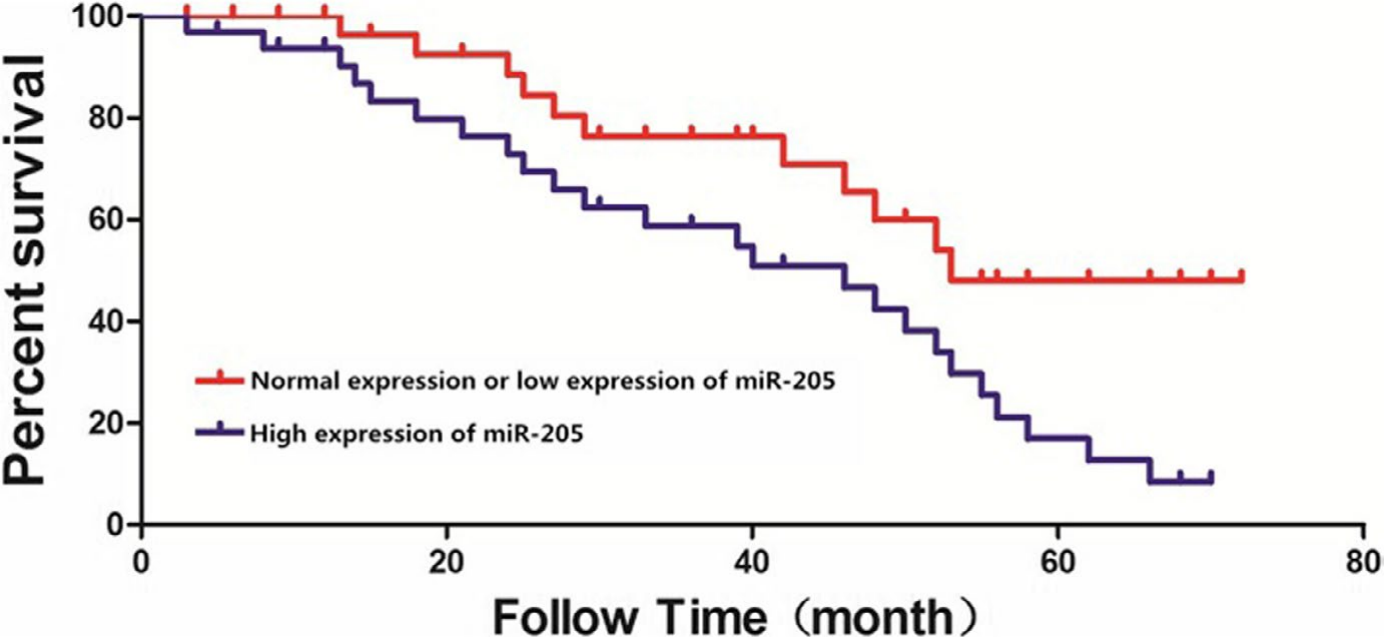
5‐year survival curves of breast cancer patients. The 5‐year survival rate of patients with low or normal expression of miR‐205‐3p is significantly higher than that in patients with high expression of miR‐205‐3p

All breast cancer patients enrolled were followed up for 5 years, the survival rate of patients was recorded, and the survival curve was drawn. According to results, the 5‐year survival rate of breast cancer patients with low or normal expression of miR‐205‐3p was significantly higher than that in patients with high expression of miR‐205‐3p (*P* < 0.01) (Figure 3).

### 3.5 Construction of cell lines with miR‐205‐3p overexpression

**Figure 4:**
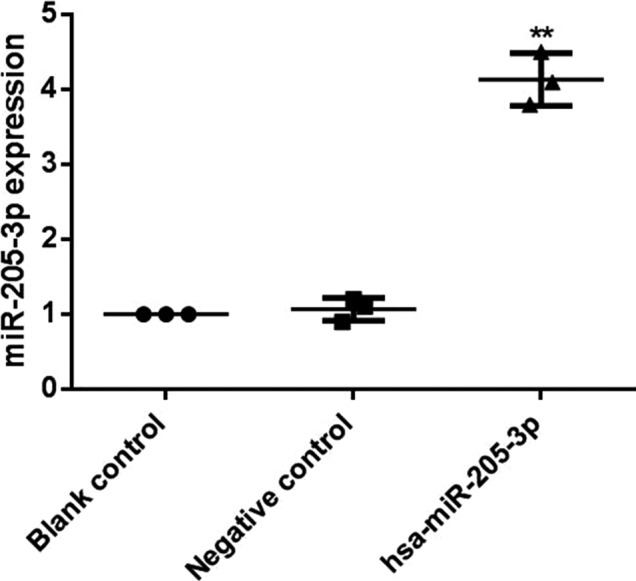
Detection of miR‐205‐3p expression after transfection by RT‐qPCR. After MCF‐7 cells were transfected with has‐miR‐205‐3p, total RNA was isolated for analysis of the expression of miR‐205‐3p by RT‐qPCR. The miR‐205‐3p expression level in cells in hsa‐miR‐205‐3p group is significantly higher than those in blank control group and negative control group, ^**^ *P* < 0.01

After human breast cancer MCF‐7 cells were transfected with miR‐205‐3p lentivirus, the miR‐205‐3p expression in each group was detected via RT‐qPCR. Results showed that miR‐205‐3p expression levels in cells in blank control group and negative control group were significantly lower than that in hsa‐miR‐205‐3p group (*P* < 0.01), indicating successful construction of MCF‐7 cell lines with miR‐205‐3p overexpression (Figure 4).

### 3.6 Effects of miR‐205‐3p on proliferation, invasion, and apoptosis of breast cancer cells

**Figure 5:**
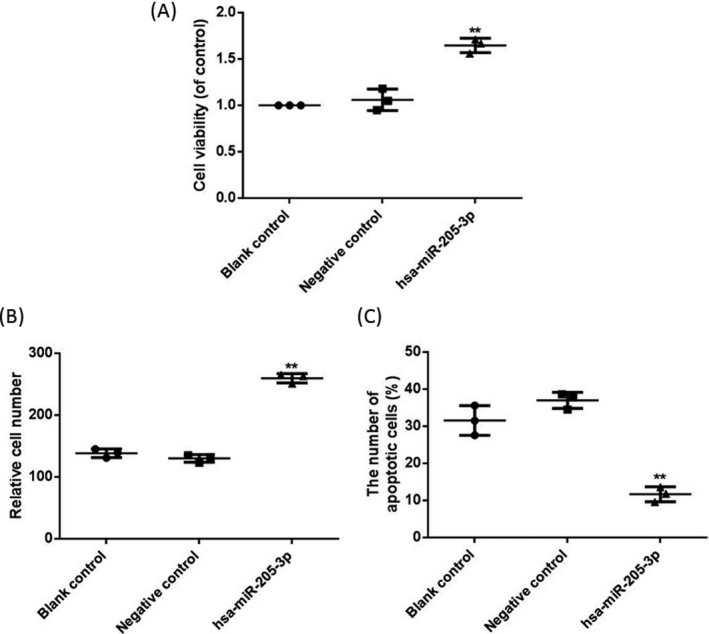
Effects of miR‐205‐3p on cell proliferation, invasion, and apoptosis. (A) Cell proliferation detected via MTT assay. (B) Cell invasion detected via transwell assay. (C) Apoptosis detected via flow cytometry. After transfection with miR‐205‐3p, both cell proliferation and invasion capacities in hsa‐miR‐205‐3p group are significantly higher than those in blank control group and negative control group, but the apoptosis level is obviously decreased, ^**^ *P* < 0.01

Effects of miR‐205‐3p on proliferation, invasion, and apoptosis of MCF‐7 cells were detected via MTT assay, transwell assay, and flow cytometry, respectively. Results showed that both cell proliferation and invasion capacities in hsa‐miR‐205‐3p group were significantly higher than those in blank control group and negative control group (*P* < 0.01, *P* < 0.01). After transfection with miR‐205‐3p, the number of apoptotic cells in hsa‐miR‐205‐3p group was significantly decreased compared with those in the blank control group and negative control group (*P* < 0.01) (Figure 5).

### 3.7 Effects of miR‐205‐3p on related protein expressions

**Figure 6:**
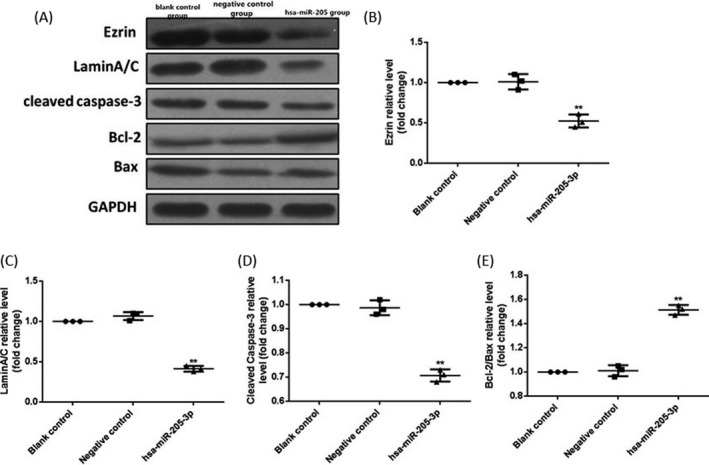
Detection of related protein expressions in MCF‐7 cells via Western blotting. (A) Protein bands. (B) Statistical graph of Ezrin protein expression level. (C) Statistical graph of LaminA/C protein expression level. (D) Statistical graph of cleaved caspase‐3 protein expression level. (E) Statistical graph of Bcl‐2/Bax protein expression level. Compared with those in the blank control group and negative control group, Ezrin, LaminA/C, and cleaved caspase‐3 expressions are remarkably lower in hsa‐miR‐205‐3p group, but the Bcl‐2/Bax ratio is higher. Data were presented as a fold change relative to GAPDH (mean ± SD). ^**^ *P* < 0.01

Ezrin, LaminA/C, cleaved caspase‐3, Bcl‐2, and Bax protein expressions in each group were detected via Western blotting. Results demonstrated that compared with those in the blank control group and negative control group, Ezrin, LaminA/C, and cleaved caspase‐3 expressions were significantly declined in hsa‐miR‐205‐3p group (*P* < 0.01), but the Bcl‐2/Bax ratio was increased significantly (*P* < 0.01) (Figure 6).

## 4 DISCUSSION

Breast cancer ranks first in the common malignant tumors in women, the current incidence of which accounts for more than 10% of the systemic tumors and seriously affects the physical health of women^19^ Breast cancer occurs frequently in postmenopausal women mainly at the age of 45‐65 years. With the environmental deterioration and other factors, a younger trend of breast cancer occurrence has been shown in recent years^20^ The radical surgical resection, combined with radiotherapy and chemotherapy, marks the improvement regarding the survival time of early breast cancer patients, but its therapeutic effect is not satisfactory on advanced breast cancer patients^21^ miRNA, as a kind of endogenous small noncoding RNA, plays an important role in cell differentiation, proliferation, and apoptosis through regulating the signaling pathway. Accumulative evidences indicate that a variety of miRNAs are involved in the regulation process of several tumors and play similar roles to tumor suppressor genes and oncogenes^22^, ^23^ The abnormal expressions of miR‐21 and miR‐205 are possibly related to the occurrence and development of breast cancer^12^ Li et al^24^ found that the miR‐205 expression level is increased in epithelial ovarian cancer, and is closely related to the proliferation and invasion of ovarian cancer. In this study, we found that the level of miR‐205‐3p was significantly upregulated in breast cancer tissues, and further in vitro assay by overexpressing miR‐205‐3p validated its role on proliferation, invasion, and apoptosis of cancer cells, which was in line with previous findings^24^.

Our data showed that Ezrin and LaminA/C protein expressions in MCF‐7 cells with miR‐205‐3p overexpression were significantly decreased compared with those in normal cells, which was consistent with previous finding^25^ Ezrin protein is an important protein molecule in the ERM protein family, which, as a connexin between cytoskeleton and cell membrane, plays an important regulatory role in cell movement and adhesion, cytoskeletal remodeling, etc Ezrin protein also exerts important effects on the occurrence and metastasis of tumors^26^ It has been demonstrated that Ezrin functions as a prognostic factor and may predict potential lung metastasis in osteosarcoma and could also possibly be used as a new therapeutic target^27^ Ezrin is closely linked with the metastatic progression of cancer and is required in tumor‐induced angio/lymphangiogenesis in vivo, which is frequently abnormally expressed in aggressive cancer types^28^ Interestingly, there is some controversy in the literature regarding the role of Ezrin in cancer metastasis and invasion. It has been indicated that the overexpression of Ezrin might promote ovarian cancer cell invasion and might predict poor prognosis^29^ However, the study revealed that patients who had negative or low Ezrin expression had a significantly shorter survival than those whose tumors had moderate or high Ezrin expression,^30^ which is consistent with our finding that Ezrin expressions were significantly declined in breast cancer tissues. Also, it has been shown that radixin, as a member of the ERM (ezrin/radixin/moesin) protein family, along with moesin and ezrin, was reduced in lung adenocarcinoma, including early‐stage bronchioloalveolar carcinoma^31^ An intriguing implication of our finding suggests that ezrin may function as tumor suppressors in breast cancer, besides lung adenocarcinoma oncogenesis, and have a distinct function in tumor cell invasion. Analysis of caveolin‐1 expression in ovarian carcinoma cell lines demonstrated DNA methylation and histone deacetylation as a means to downregulate caveolin expression^32^ The similar mechanisms may reduce ezrin expression, in addition to genetic alterations associated with breast carcinogenesis.

Expression of A‐type lamins increases the risk of death from colorectal cancer because its presence gives rise to increased invasiveness but not affect cell proliferation^33^ By contrast, previous data indicate that low expression of lamin A/C is associated with an increased disease recurrence in stage II and III colon cancer patients^34^ Moreover, evidence unraveled that cultured breast cancer cell lines expressed less lamin A/C compared with noncancerous mammary gland cells, which is in line with our result that LaminA/C was significantly decreased in breast cancer tissues^35^ The loss of lamin A/C expression is associated with variables of poor prognosis and shorter outcome in patients with breast cancer^36^ The loss of A‐type lamins was shown to induce changes in nuclear distribution of telomeres and to affect telomere function, thereby inducing chromosomal instability^37^ The malfunction of A‐type lamins will increase chromosomal instability and stimulate carcinogenesis. Increasing genomic instability may enhance tumor progression, including generation of tumor cell clones that have the capacity to cause disease recurrence.

Mitochondrial pathway plays an important role in mediating the apoptosis process. Antiapoptotic protein Bcl‐2, proapoptotic protein Bax, and downstream cleaved caspase‐3 are involved in apoptosis cascade reaction and apoptosis of tumor cells^38^ Both in vitro and in vivo experiments in this study manifested that the increased miR‐205‐3p expression led to the significant decrease in the cleaved caspase‐3 expression, significant increase in the Bcl‐2/Bax ratio in cells, thus reducing the apoptosis level.

Wei et al^39^ studied and found that miR‐205 has close correlations with clinical progression and prognosis of ovarian cancer patients. In this study, correlations of miR‐205‐3p expression in tumor tissues with clinicopathological conditions and recurrence of breast cancer patients were analyzed. According to results, the miR‐205‐3p expression was related to the size, TNM staging, metastasis, and recurrence of tumor, and the 5‐year survival rate of patients with high expression of miR‐205‐3p was obviously lower than that of patients with normal or low expression. Moreover, miR‐205‐3p was identified as an independent factor for the survival of breast cancer patients. Our data demonstrate that correlation of miR‐205‐3p with prognosis of breast cancer patients was established in those patients that died because the breast cancer but no other causes and miR‐205‐3p may serve as an important gene for prognosis evaluation of breast cancer. The limitation in our study still exists that the controversial result regarding the changes in Ezrin, LaminA/C in tumor tissues, and the potential mechanisms remain to be further investigated. We propose that cancer cells exhibit a variety of features indicative of atypical nuclei, while the changes in these indicators may be further related to tumor's pathological classification and cancer progression.

In conclusion, significantly higher miR‐205‐3p expression is found in breast cancer tissues and associated with shorter survival of patients. Highly expressed miR‐205‐3p promotes the proliferation and invasion and reduces the apoptosis of breast cancer cells in vitro, suggesting therapeutic targeting miR‐205‐3p might be a novel approach for the treatment of breast cancer.
